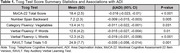# The Intersection of Cognitive Function, Hypertension, and Social Determinants of Health: Insights from a Large, Multicenter Trial

**DOI:** 10.1002/alz70860_107742

**Published:** 2025-12-23

**Authors:** Adam Parks, Xing Song, Kate Young, Jonathan D Mahnken, Sravani Chandaka, Diego R Mazzotti, Molly Conroy, Mark A. Supiano, Jeffrey M. Burns, Aditi Gupta

**Affiliations:** ^1^ University of Kansas Medical Center, Kansas City, KS, USA; ^2^ University of Missouri School of Medicine, Columbia, MO, USA; ^3^ University of Utah, Salt Lake City, UT, USA

## Abstract

**Background:**

Uncontrolled hypertension, common among older adults, has been shown to increase the risk of cognitive impairment. Hypertension management, crucial for public health, is often hindered by environmental factors known as social determinants of health (SDOH). Individuals in disadvantaged SDOH conditions face a higher risk of uncontrolled hypertension due to social and environmental stressors, emphasizing the need for targeted interventions in these communities. This study examined how individual health factors, including blood pressure (BP) and cognitive function, relate to population‐level environmental factors in a large, multicenter trial.

**Method:**

Baseline BP and cognition were measured during a baseline visit with patients enrolled in a remote‐based hypertension management clinic. The association between cognitive function and the Area Deprivation Index (ADI), a multidimensional measure of a region's socioeconomic status linked to health outcomes, was analyzed. The ADI score ranges from 1 to 100, with higher scores indicating greater deprivation. Cognition was assessed using the Telephone Cognitive Assessment (Tcog) neuropsychological battery. Multiple regression analyses, that included age, sex, race, and systolic BP as covariates, were conducted to assess these associations.

**Result:**

The final sample included 1000 patients with a mean (SD) age of 73.3 (5.8) and baseline SBP of 135 (17) mmHg; 61% were female, 88% were White, 10% were Black and 2% were Hispanic. The median (IQR) ADI of the cohort was 34 (18,55). Higher ADI ranking was significantly associated with lower global cognitive function (MoCA‐22) (β = ‐0.016, 95% CI: ‐0.023 to ‐0.01, *p* < 0.001) as well as lower scores in memory (Rey Auditory Verbal Learning Test Delayed Recall), attention (Number Span Backward), and language (Category Fluency: Vegetables, Phonemic Fluency F and L Words) (see Table 1).

**Conclusion:**

Socioeconomic deprivation, as measured by a higher ADI, is associated with lower performance across multiple cognitive domains, independent of age, sex, race, and systolic BP. These findings highlight the importance of addressing the impact of SDOH on cognition in hypertension management. Future research will explore whether targeted interventions, such as remote hypertension management programs, can reduce these disparities and improve cognitive outcomes in socioeconomically disadvantaged areas.